# A randomized, placebo-controlled phase 2 study of paclitaxel in combination with reparixin compared to paclitaxel alone as front-line therapy for metastatic triple-negative breast cancer (fRida)

**DOI:** 10.1007/s10549-021-06367-5

**Published:** 2021-09-03

**Authors:** Lori J. Goldstein, Mauro Mansutti, Christelle Levy, Jenny C. Chang, Stephanie Henry, Isaura Fernandez-Perez, Jana Prausovà, Elzbieta Staroslawska, Giuseppe Viale, Beth Butler, Susan McCanna, Pier Adelchi Ruffini, Max S. Wicha, Anne F. Schott, for the fRida Trial Investigators, Ricardo H. Alvarez, Anne F. Schott, Maysa Abu-Khalaf, Nuhad Ibrahim, Brooke Daniel, Michael Meshad, David Kanamori, Amelia Zelnak, Mark Graham, Jason Comer, Manon Huizing, Francois Duhoux, Vincent Richard, Didier Verhoeven, Martin Smakal, Marta Krasenska, Milan Kohoutek, Martina Zimovjanova, Eugen Kubala, Mario Campone, Jean-Marc Ferrero, Anthony Goncalves, Laurence Venat-Bouvet, Jacques Medioni, Laura Biganzoli, Hector Soto Parra, Paolo Pedrazzoli, Marco Colleoni, Mauro Moroni, Dino Amadori, Paolo Morandi, Saverio Cinieri, Piotr Tomczak, Tomasz Sarosiek, Marek Wojtukiewicz, Andrzej Mruk, Bożena Kukielka-Budny, Silvia Antolin Novoa, Estela Vega Alonso, Miguel Martin Jimenez

**Affiliations:** 1 Department of Medical Oncology, Fox Chase Cancer Center, 333 Cottman Ave, Philadelphia, PA 19111 USA; 2 Azienda Ospedaliero Universitaria Santa Maria della Misericordia, 33100 Udine, Italy; 3 CLCC Francois Baclesse, Caen, France; 4 The Methodist Hospital Research Institute, Houston, Tx 77030 USA; 5 CHU UCL Namur, site Ste Elisabeth, Namur, Belgium; 6 Hospital Alvaro Cunqueiro, 36204 Vigo, Spain; 7 Fakultni nemocnice v Motole, Onkologická klinika 2, LF UK a FN Motol, Praha, Czech Republic; 8 Centrum Onkologii Ziemi Lubelskiej św. Jana z Dukli, Lublin, Poland; 9 IEO Istituto Europeo di Oncologia IRCCS, 20141 Milano, Italy; 10 University of Milan, Milano, Italy; 11 Research and Development, Dompé farmaceutici s.p.a., 20122 Milano, Italy; 12 Rogel Cancer Center, University of Michigan, Ann Arbor, MI 48109 USA

**Keywords:** TNBC, Cancer stem cells, Reparixin, CXCR1

## Abstract

**Purpose:**

CXCR1, one of the receptors for CXCL8, has been identified as a druggable target on breast cancer cancer stem cells (CSC). Reparixin (R), an investigational oral inhibitor of CXCR1, was safely administered to metastatic breast cancer patients in combination with paclitaxel (P) and appeared to reduce CSC in a window-of-opportunity trial in operable breast cancer. The fRida trial (NCT02370238) evaluated the addition of R to weekly as first-line therapy for metastatic (m) TNBC.

**Subjects and Methods:**

Subjects with untreated mTNBC were randomized 1:1 to R or placebo days 1–21 in combination with weekly P 80 mg/m^2^ on days 1, 8, 15 of 28-day cycles. The primary endpoint was PFS by central review.

**Results:**

123 subjects were randomized (62 to R + P and 61 to placebo + P). PFS was not different between the 2 groups (median 5.5 and 5.6 months for R + P and placebo + P, respectively; HR 1.13, *p* = 0.5996). ALDH^+^ and CD24^−^/CD44^+^ CSC centrally evaluated by IHC were found in 16 and 34 of the 54 subjects who provided a metastatic tissue biopsy at study entry. Serious adverse events (21.3 and 20% of subjects) and grade ≥ 3 adverse reactions (ADR) (9.1 and 6.3% of all ADRs) occurred at similar frequency in both groups.

**Conclusion:**

fRida is the first randomized, double-blind clinical trial of a CSC-targeting agent in combination with chemotherapy in breast cancer. The primary endpoint of prolonged PFS was not met.

**Clinical Trial Registration/Date of Registration:**

NCT01861054/February 24, 2015.

**Supplementary Information:**

The online version contains supplementary material available at 10.1007/s10549-021-06367-5.

## Introduction

Cancer stem cells (CSC) have the ability to self-renew and generate the full range of cells that make up a bulk tumor. Experimental models and retrospective clinical observations point to CSC as responsible for tumor initiation, treatment resistance, disease recurrence, and metastasis [1]. An ideal CSC-targeting agent should be a non-toxic molecule that can be safely administered also in combination with chemotherapy to improve disease control over non CSC, bulk tumor cells.

Breast cancer was the first solid tumor where CSC were identified [2]. Two markers are commonly used to identify such cells in clinical specimens, i.e., aldehyde dehydrogenase (ALDH) and CD24/CD44. CXCR1, one of the receptors for CXCL8, has been identified on breast cancer ALDH + CSC [3]. Binding of CXCL8 to CXCR1 on the CSC surface protects CSC from pro-apoptotic signals released in the tumor microenvironment following taxane administration [4]. A CXCL8–CXCR1 axis in breast cancer CSC heightened by taxane administration has been reported by several independent laboratories [5–7]*.*

Reparixin, an investigational allosteric inhibitor of CXCR1, reduced CSC in breast cancer (BC) xenografts both as single agent and in combination with taxane chemotherapy [4]. In a phase Ib trial in women with metastatic HER2-negative BC, the combination of escalating doses (400 to 1200 mg three times per day) of reparixin with weekly paclitaxel resulted in a low incidence and severity of adverse reactions, a sizeable response rate and time-to-progression, with some long-term responders [8]. Furthermore, in a window-of-opportunity clinical trial, a 21-day course of reparixin before curative surgery appeared to reduce CSC by flow cytometric analysis in several subjects [9].

Patients with metastatic triple-negative breast cancer (TNBC) receiving single-agent chemotherapy have poor clinical outcomes with median overall survival of around 18 mos. or less [10, 11]. Considering the CSC enrichment/signature of TNBC [12, 13], a phase 2, randomized, double-blind study [fRida (NCT02370238)] evaluating the safety and efficacy of reparixin plus paclitaxel *vs.* placebo plus paclitaxel in untreated metastatic (m) TNBC was initiated.

## Materials and methods

### Oversight

The trial was conducted according to GCP and the principles of the Declaration of Helsinki. All the subjects provided written informed consent. Protocol approval was obtained from Independent Review Boards or Ethics Committee at each site. An independent DMC reviewed unblinded safety data every 6 months. All the authors verify that the trial was conducted according to the protocol and vouch for the accuracy and completeness of the data. All the drafts of the manuscript were prepared by the authors. The study agent, reparixin, and placebo were provided by the study sponsor, Dompe, who also worked collaboratively with the study investigators for study design, data collection, analysis, and interpretation.

### Subjects

Eligible subjects were female 18 years of age or older and had stage IV, histologically documented TNBC according to ASCO-CAP guidelines [14, 15], as evaluated by local institutions. They were eligible to receive paclitaxel monotherapy and had received no prior systemic therapy for advanced disease. de novo stage IV patients were allowed to be randomized only following protocol amendment nr. 2 in the second half of 2016. Radiation therapy and (neo)adjuvant chemotherapy (including taxanes) were allowed if treatment was completed ≥ 12 and ≥ 6 months for taxane and non taxane regimens, respectively. Measurable disease according to RECIST 1.1 an ECOG performance status ≤ 1 and an adequate hematologic and organ function were also required. Main exclusion criteria were brain metastases (a baseline CT or MRI of the brain was mandatory) and G > 1 peripheral neuropathy. The full eligibility criteria are provided in the protocol, available with the full text of this article at https://www.springer.com/journal/10549.

### Trial design and procedures

Subjects were randomly assigned in a 1:1 ratio with an interactive voice–web response system to receive paclitaxel in combination with either reparixin or placebo. Stratification factor was the history or not of (neo)adjuvant chemotherapy. Subjects received paclitaxel 80 mg/m^2^ on days 1, 8, and 15 and reparixin/placebo oral tablets 1200 mg t.i.d. from day 1 to 21 of 28-day cycles. Subjects received study drugs until disease progression according to RECIST 1.1, withdrawal of consent or unacceptable toxicity, whichever occurred first.

The discontinuation of either reparixin/placebo or paclitaxel and continuation of treatment with either one as single agent was not allowed. Prespecified modifications of the paclitaxel dose were permitted in order to manage the side effects of chemotherapy.

Tumor imaging occurred at baseline and every 8 weeks. Patient management was based upon local radiologist evaluation. Follow-up for survival occurred every 3 months after discontinuation of study treatment.

The primary endpoint was progression-free survival (PFS) as determined by blinded independent radiology review (IRR). Secondary endpoints were overall survival (OS), objective response rate (ORR), and safety of the combination. The primary and secondary efficacy analyses were evaluated primarily for the intention-to-treat (ITT) population, which included all the subjects who had undergone randomization whether or not they received study drugs. The safety population consisted of all subjects who received at least one dose of study treatment.

Exploratory endpoints were time to new metastasis (TTM) and proportion of subjects progressing with new metastatic lesions (both at pre-existing and new sites), the measurement of CSC in metastatic tissue samples, and the incidence and severity of paclitaxel-induced peripheral neuropathy. To this purpose, subjects progressing with no new systemic metastatic lesions were requested to undergo brain imaging to rule out subclinical CNS lesions.

The safety population included all subjects who took at least one dose of the study treatment and was evaluated according to CTCAE version 4.03. Additional details regarding the study design, including key protocol amendments, are available with the protocol.

### CSC

A formalin-fixed paraffin-embedded sample of metastatic tissue was obtained, whenever feasible, for prospective centralized measurement/evaluation of CSC. ALDH1 was immunostained using the mouse monoclonal antibody 44/ALDH (Becton, Dickinson & Co, Franklin Lakes, NJ, USA) at a 1:200 dilution, for 30 min at room temperature, followed by incubation with the Bond Polymer Refine Detection Kit (Leica Biosystems, Buccinasco, Italy) on a Bond platform (Leica).

Dual staining for CD44 and CD24 was performed on 3 um thin sections pre-treated with a 10 mM citrate pH 6.0 solution at 98 °C for 30 min followed by a cooling time of 20 min at room temperature.

Sections were then incubated with the primary antibody cocktail (mouse IgG1 anti-Human CD44v6, clone VFF-18, at 1:250 dilution and mouse IgMk anti-Human CD24, clone SN3b, 1:25, Invitrogen, Carlsbad, CA, USA) in a humidity chamber at 4 °C overnight and 1 h at room temperature. Sequential detection steps were performed with Biotin-conjugated Rat anti-Mouse IgG1 (clone M1-14D12, 1:50, eBioscienceTM-Invitrogen) and Streptavidin Protein DyLight 488 (1:200, ThermoFisher Scientific, Waltham, MA, USA) for CD44, and with Alexa Fluor 546-conjugated Goat anti-Mouse IgM (1:250, ThermoFisher Scientific) for 30 min at room temperature. Finally, sections were mounted with Vectashield Antifade Mounting Medium with DAPI (ready to use, Vector Lab, Burlingame, CA, USA) and a coverslip sealed with nail polish. The slides were stored at − 20 °C until screening with a Zeiss AxioImager M2 Microscope, equipped with fluorescence filters DAPI (350/50-460/50), Green (490/40-537/29), and Orange (546/12-590/33), using the Metafer Slide Scanning System software (MetaSystems GmbH, Altlussheim, Germany).

### Statistical analysis

The trial was initially designed to randomly assign 156 subjects for the evaluation of a primary endpoint of PFS. 142 PFS events were required to provide 80% power to detect a difference in median PFS from 5 to 8 months corresponding to a hazard ratio of 0.625, when using a logrank statistic having (one-sided) 0.025 false positive error rate.

Due to extreme enrollment difficulties during the first 6 months of 2018, accrual of subjects to the study was terminated early (July 30, 2018) and the final sample size is 123 randomized subjects. No formal recalculation of sample size/required PFS events could be made under these circumstances and the above assumptions remained.

PFS and OS were compared between treatment arms using stratified log-rank test. Hazard ratios for disease progression and death were estimated with the use of a Cox proportional model stratified by randomized sub-group. Kaplan–Meier curves were produced to estimate median PFS, median OS, and median TTM outcomes. Similar methods were applied to the duration of response.

The comparisons of the response rate and the proportion of subjects progressing with new metastatic lesions were made with the use of the Cochran–Mantel–Haenszel (CMH) test, stratified by the patient population (newly diagnosed vs. relapsed).

The CSC markers (CD24^−^CD44^+^ and ALDH^+^ assessed by IHC) within the epithelial cell population were summarized by descriptive statistics (n, mean, standard deviation, median, minimum, and maximum).

The statistical analyses were carried out with SAS version 9.4 (or later versions) from the SAS Institute.

## Results

**Fig. 1:**
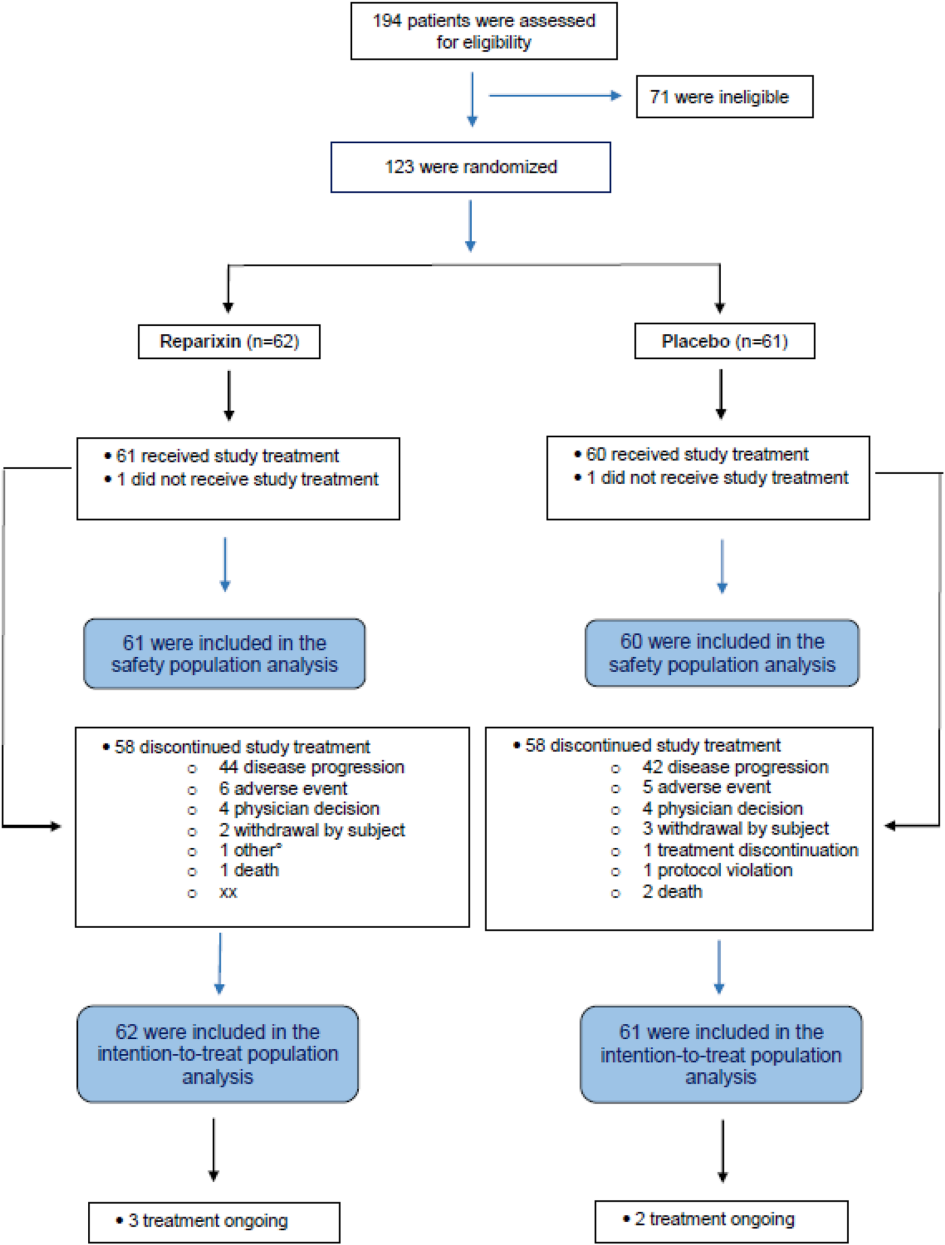
Patient disposition: Randomization, trial populations, and follow-up are shown, and so are the numbers of subjects who were receiving study treatment on the data cutoff date (February 20, 2019)

From July 2015 to May 2018, 194 subjects were assessed for eligibility and 123 (ITT population) were randomized to either reparixin (n = 62) or placebo (n = 61) at 47 clinical sites in Europe and USA. A total of 87 subjects were randomized in Europe and 36 in USA. One patient in each group did not receive study treatment and was excluded from the safety population. The disposition of subjects is depicted in Fig. 1. Overall, the characteristics of the subjects at baseline were well balanced between the two treatment groups (Table 1).

**Table 1:** Characteristics of the subjects at baseline

Characteristic	ITT	
	Reparixin + Paclitaxel (*N* = 62)	Placebo + Paclitaxel (*N* = 61)
Age		
Median (range)	57 (29–79)	57.5 (33–77)
Distribution—no (%)		
≤ 40 years	5 (8.0%)	10 (16.4%)
41–64 years	40 (64.5%)	35 (57.4%)
≥ 65 years	17 (27.4%)	16 (26.2%)
Race or ethnic group		
White	46 (74.2%)	49 (80.3%)
Asian	0	1 (1.6%)
Black or African American	6 (9.7%)	7 (11.5%)
Not collected per local requirements	10 (16.1%)	4 (6.5%)
ECOG PS score—no. /total no. (%)		
0	38 (61.3%)	41 (68.3%)
1	24 (39.3)	20 (32.8%)
No. of sites of metastatic disease		
Visceral disease	44 (71.0%)	51 (83.6%)
Site of metastatic disease		
Liver—no. (%)	20 (32.2%)	19 (31.1%)
Lung—no. (%)	33 (53.2%)	37 (60.6%)
Bone—no. (%)	21 (33.9%)	24 (39.3%)
Lymph node only—no. (%)	3 (4.8%)	3 (4.9%)
Previous therapy—no. (%)		
(Neo)adjuvant therapy	48 (77.4%)	53 (86.9%)
Taxane		
Yes	40 (64.5%)	45 (73.8%)
No	7	8
de novo stage IV	14 (22.6%)	8 (13.1%

*ECOG PS* eastern cooperative oncology group performance status

At the clinical cutoff date (February 20, 2019), for subjects in the reparixin–paclitaxel group, the median duration of reparixin and paclitaxel treatment was 16.6 and 16.1 weeks, respectively. For subjects in the placebo–paclitaxel group, the median duration of placebo and paclitaxel treatment was 15.5 and 14.7 weeks, respectively. The mean (± SD) cumulative dose of paclitaxel was 1211.7 ± 944.58 mg/m^2^ in the reparixin–paclitaxel group and 1344.2 ± 1090.35 mg/m^2^ in the placebo–paclitaxel group.

Palliative radiation therapy was administered in 1 patient in the reparixin group and in 2 subjects in the placebo group.

### Efficacy

**Fig. 2:**
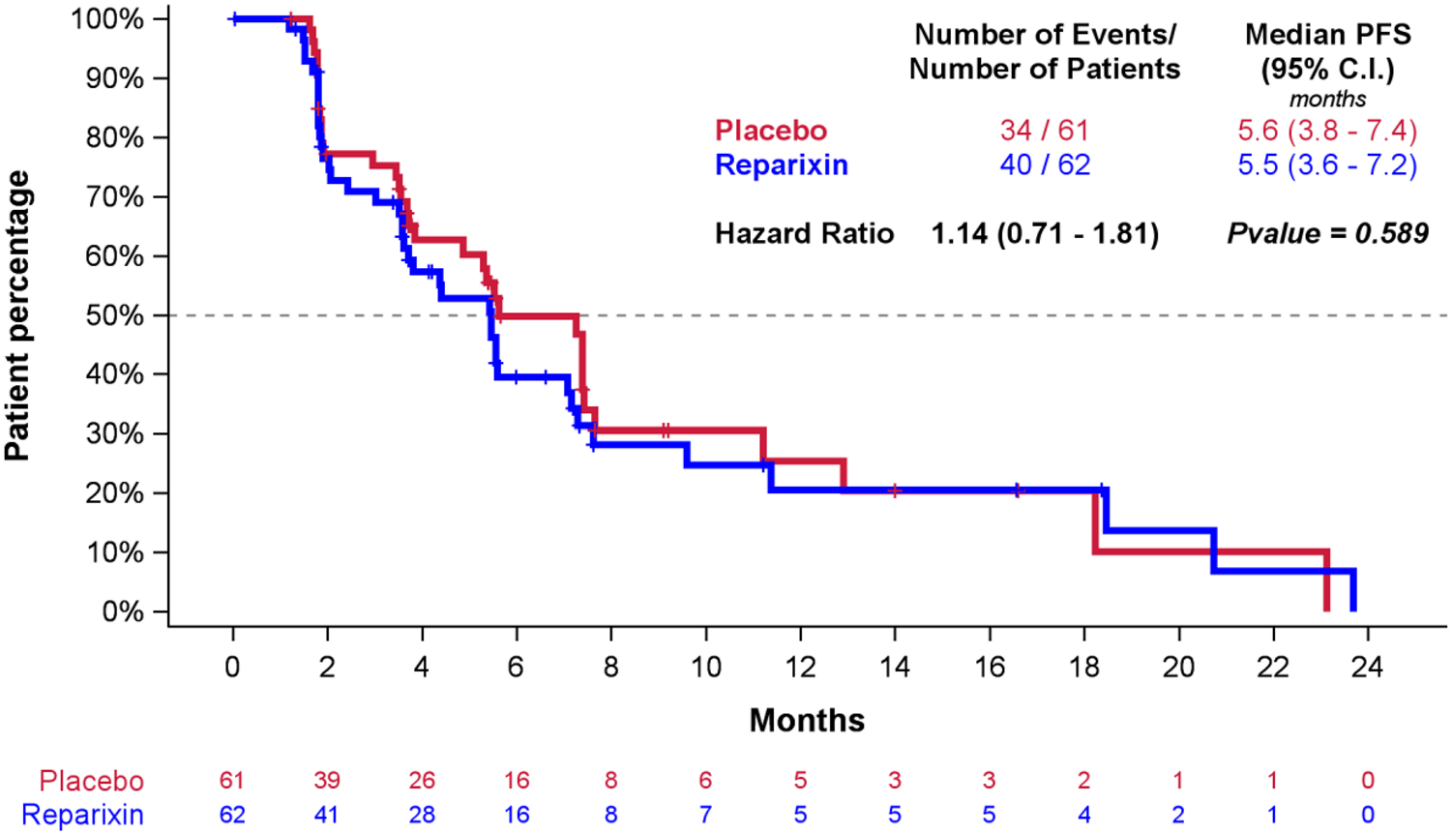
Progression-free survival in the ITT population: Kaplan–Meier estimates of PFS, according to the Response Evaluation Criteria in Solid Tumors, version 1.1, as assessed by the independent radiology review, among subjects in the ITT population. Stratified hazard ratios for disease progression or death are reported along with *p* values. Tick marks indicate censored data

At the time of data cutoff, the median follow-up was 14.3 months in the ITT population (14.3 months in the reparixin–paclitaxel arm and 12.9 months in the placebo–paclitaxel arm) and 92 PFS events had been reported by investigators. Progression-free survival by IRR after 74 events (40 in reparixin and 34 in placebo group) was not significantly different between the two treatment groups (median, 5.5 vs. 5.6 months in reparixin–placebo and placebo–paclitaxel arm, respectively; stratified hazard ratio 1.13; 95% confidence interval [CI] 0.71–1.80; *p* = 0.5996) (Fig. 2). Also, PFS assessed by investigators did not show differences between treatment groups (median 5.5 and 5.8 months in reparixin–paclitaxel and placebo–paclitaxel group, respectively; HR 1.12; 95% CI 0.74–1.70; *p* = 0.578). Subgroup analyses (i.e., visceral disease, prior taxane, age < 40 years) did not differ from the main analysis (data not shown).

**Fig. 3:**
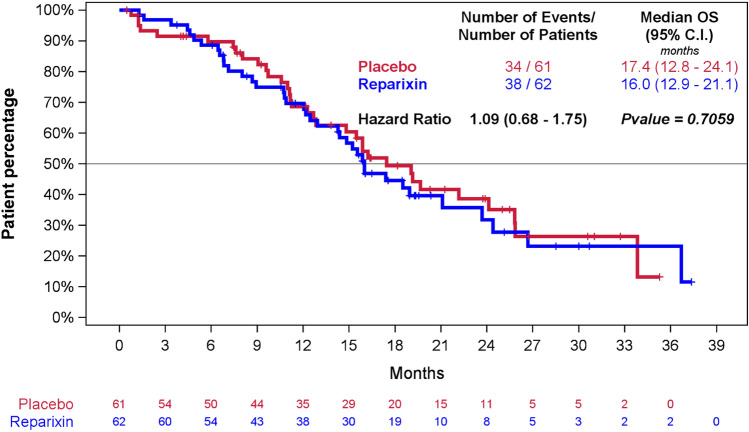
Overall survival in the ITT population: Kaplan–Meier estimates of OS among subjects in the ITT population. Stratified hazard ratios for death are reported along with *p* values. Tick marks indicate censored data. 6 subjects (4 in reparixin + paclitaxel and 2 in placebo + paclitaxel) did not re-sign ICF to protocol amendment 2 or later and were censored at 1 year after the off treatment visit

Subsequent anticancer therapy was administered to 45 patients (73.8%) in the reparixin–paclitaxel group and to 40 (66.7%) in the placebo–paclitaxel arm and was generally balanced between the two groups (Table S1). At the time of data cutoff, 38 of 62 subjects (61.3%) in the reparixin–paclitaxel arm and 34 of 61 (55.7%) in the placebo–paclitaxel arm had died. The median overall survival was 16.0 and 17.4 months in the reparixin–paclitaxel and the placebo–paclitaxel arm, respectively (stratified hazard ratio for death 1.09; 95% CI 0.68–1.75; p = 0.7059] (Fig. 3).

In the ITT population, the rate of confirmed objective responses, as assessed by blinded IRR on the ITT population, was 25.8% (95% CI 0.17–0.42) and 22.9% (95% CI 0.15–0.40) in the reparixin–paclitaxel and in the placebo–paclitaxel group, respectively. Only 1 subject, in the reparixin + paclitaxel group, experienced a complete response. The median duration of response (DOR) in the ITT as assessed by IRR was 9.8 (95% CI 3.8–16.8) months in the reparixin–paclitaxel arm and 5.7 (95% CI 3.7–14.8) months in the placebo–paclitaxel arm (*p* = 0.767).

Time to new metastasis was not different between treatment arms (data not shown). In the ITT population, the number of subjects who at progression displayed new metastatic lesions at existing or new sites was 17/40 (42.5%) in the reparixin–paclitaxel arm and 23/34 (67.6%) in the placebo–paclitaxel arm (p = 0.0305, Chi-square test). A similar proportion of subjects developed brain metastases (data not shown).

### Safety

Among subjects in the safety population, treatment-emergent adverse events (TEAE) occurred in 60 (98.4%) and 57 (95%) subjects in the reparixin and placebo arm, respectively. The most common TEAE in the reparixin + paclitaxel group were nausea (37.7% of subjects), alopecia (34.4%), anemia (29.5%), diarrhea (26.2%), and asthenia (26.2%), while in the placebo + paclitaxel group were fatigue (43.3%), nausea (36.7%), alopecia (35%), diarrhea (25.0%), and asthenia (21.7%). Serious TEAEs occurred in 13 (21.3%) and 12 (20%) subjects in the reparixin and placebo group, respectively.

Adverse events that led to withdrawal of study treatment occurred in 7 (11.5%) and 13 (21.7%) subjects in reparixin and placebo arm, respectively.

Fatal TEAE occurred in 3 (4.9%) and 4 (6.7%) subjects in reparixin and placebo group, respectively. One death in the reparixin arm (from peritonitis and intestinal perforation) was considered by the investigators to be possibly related to the study treatment.

The frequency and severity of peripheral neuropathy was similar between the 2 groups (29.5% and 30.0% in reparixin and placebo arm, respectively). However, events grade 2 or greater were more common in placebo group (6.5% with reparixin vs. 16.6%, *p* = 0.0822 chi-squared test). Two subjects in reparixin + paclitaxel group and 3 subjects in placebo + paclitaxel group discontinued study treatment due to TEAE of peripheral neuropathy.

Interestingly, a statistically significant difference was observed for the TEAE fatigue, which was recorded in 11 (18%) and 26 (43.3%) subjects in the reparixin and placebo group, respectively (*p* = 0.003, Chi-square test). Also, G3 fatigue was recorded only in 2 subjects in the placebo group. 0 and 3 subjects in the reparixin and placebo group, respectively, discontinued study treatment due to TEAEs including asthenia.

A higher number of ADR (as assessed by the investigator) were recorded in subjects receiving R + P (263) compared to subjects receiving placebo + P (200). The percentage of grade ≥ 3 ADR was similar between the two groups (9.1 and 6.0% recorded in 14 and 6 subjects in reparixin and placebo arm, respectively). Two and 0 subjects experienced G > 3 ADRs in reparixin and placebo arm, respectively. The most common adverse reactions are presented in Table 2.

**Table 2:** Summary of adverse drug reactions in the safety population

	Reparixin + Paclitaxel (*n* = 61)		Placebo + Paclitaxel (*n* = 60)	
	Any grade	Grade ≥ 3	Any grade	Grade ≥ 3
	*n* (%)		*n* (%)	
Nausea	15 (24.6)	2 (3.3)	16 (26.7)	1 (1.7)
Diarrhea	9 (14.8)	0	7 (11.7)	0
Vomiting	8 (13.1)	1 (1.6)	2 (3.3)	0
Asthenia	7 (11.5)	0	12 (20.0)	0
Fatigue	7 (11.5)	0	17 (28.3)	2 (3.3)
Headache	7 (11.5)	0	2 (3.3)	0
Anemia	7 (11.5)	0	1 (1.7)	0
Dysgeusia	6 (9.8%)	0	2 (3.3%)	0
Alopecia	5 (8.2)	0	9 (15.0)	0
Constipation	5 (8.2%)	0	4 (6.7%)	0
Paresthesia	4 (6.6%)	0	3 (5.0%)	0
Abdominal pain	3 (4.9%)	0	4 (6.7%)	0
Abdominal pain upper	3 (4.9%)	0	4 (6.7%)	0
Neutropenia	5 (8.2%)	2 (3.3%)	5 (8.3%)	4 (6.7%)
Arthralgia	3 (4.9%)	0	3 (5.0%)	0
Myalgia	3 (4.9%)	0	3 (5.0%)	0
ALT elevation	4 (6.6%)	1 (1.6%)	2 (3.3%)	1 (1.7%)
AST elevation	4 (6.6%)	1 (1.6%)	2 (3.3%)	1 (1.7%)
Decreased appetite	2 (3.3%)	0	3 (5.0%)	0
Peripheral neuropathy	2 (3.3%)	0	3 (5.0%)	0
Dyspepsia	2 (3.3%)	0	4 (6.7%)	0
Stomatitis	1 (1.6%)	0	3 (5.0)	0
Rash	7 (11.5)	0	3 (5.0)	0
Gastro esophageal reflux	0	0	3 (5.0%)	0

Adverse drug reactions in 5% or more subjects (any grade) in either treatment group; worst grade reported (eg., a patient who had an event at both grade 3 and 4 appears only in the grade 4 column)

### Cancer stem cells

CSC analysis was performed on metastatic tumor tissue only. An evaluable biopsy of metastatic tissue was obtained at baseline from 54 randomized patients (Table 3). ALDH-1^+^ and CD24^−^/CD44^+^ cells were detected within the epithelial tumor cell populations in 16 and 34 patients, respectively. Only in 9 biopsies, CSC of both phenotype could be detected. Since the presence of CSC was not a stratification factor, subjects with positive biopsies for CSC markers were unevenly distributed in the two treatment groups, i.e., 12 vs. 4 and 22 vs. 12 for ALDH^+^ and CD24^−^/CD44^+^ in reparixin and placebo group, respectively. When PFS was analyzed by the presence or absence of CSC markers (i.e., ALDH^+^ or CD24^−^/CD44^+^ cells), no difference between groups was observed (data not shown). In a *post hoc* analysis on subjects with ALDH^+^ CSC, PFS and OS appeared to be longer in reparixin than in placebo group (Figure S1) and so did OS in subjects with CD24^−^/CD44^+^ CSC (Figure S2); however, the overlapping 95% CI and the small number of patients in each group do not allow to draw any conclusion.

**Table 3:** Cancer stem cells in metastatic tumor tissue

	Reparixin + Paclitaxel No. + /total no. with biopsy (%)	Placebo + Paclitaxel No. + /total no. with biopsy (%)	Total
ALDH^+^	12/31 (38.7%)	4/23 (17.4%)	16/54 (29.6%)
≤ 1%	4	2	6
> 1 ≤ 5%	2	0	2
> 5%	6	2	8
ALDH intensity			
1	2	1	3
2	5	3	8
3	5	0	5
CD24^−^/CD44^+^	22/31 (71%)	12/23 (52.2%)	34/54 (62.9%)
≤ 1%	5	3	8
> 1 ≤ 5%	0	0	0
> 5%	17	9	26
ALDH^+^ and CD24^−^/CD44^+^	8/31 (25.8%)	1/31 (3.2%)	9/54 (16.6%)

Cancer stem cells as evaluated by central pathology by immunohistochemistry. Tissue sections were either stained for ALDH-1 or double stained for CD24 and CD44

## Discussion

Herein, we report on the first randomized, double-blind, placebo-controlled clinical trial of a CSC-targeting agent in breast cancer. Administered as first-line treatment, the combination of reparixin and paclitaxel did not improve PFS of mTNBC patients over paclitaxel alone. The median PFS in both groups was within the range reported in other contemporary trials in first-line mTNBC treated with single-agent taxanes [16, 17], with the exception of the PAKT trial [18]. Although immature, OS data also did not show a difference between treatment groups.

From a safety standpoint, the combination appeared to be well tolerated and although patients receiving R + P experienced a higher number of adverse reactions, the proportion of severe ADR was similar in the two groups. Following up on preclinical evidence [19] and clinical suggestion [8] of a protective effect of reparixin on paclitaxel-induced peripheral neuropathy, a trend toward a preventive effect of reparixin on peripheral neuropathy grade 2 or greater was observed. However, the trial was not designed to specifically address this question and it is underpowered for the limited number of events recorded. Interestingly, analysis of TEAE revealed that administration of reparixin may lead to a reduction in incidence and severity of cancer-related fatigue. A possible role for IL-8 in CRF has been reported in several studies [20, 21].

Cancer stem cells were the target of reparixin activity, through inhibition of CXCR1 [4]. At study entry, only 54/123 patients provided an evaluable biopsy of metastatic tissue, thus limiting the possibility to fully explore the role of CSC as a therapeutic target in this setting.

Furthermore, only a proportion of subjects’ tumors stained positive for either CSC population and positive biopsies were unevenly distributed in the treatment groups, limiting the power of the study to determine the predictive value of these markers.

Considering the challenges posed by either obtaining serial biopsies of metastatic tissue during treatment [22] or enumerating CSC in peripheral blood [8], anti-CSC activity was also evaluated by means of time to new metastasis and proportion of subjects progressing with new metastatic lesions [23, 24]. The rationale behind this exploratory analysis is that disease progression by enlargement of pre-existing lesions is mainly reflective of therapeutic effects on bulk tumor cell populations, while detection of new lesions may reflect treatment effects on CSC’s. In ITT population, no difference was observed between reparixin and placebo in terms of time to new metastasis. Although a numerically lower proportion of subjects progressed with new lesions in the reparixin–paclitaxel as compared to the placebo–paclitaxel group, this did not reach statistical significance.

The negative results reported for several clinical trials of CSC-targeting agents have called into question on the clinical utility of targeting CSC [25, 26]. Indeed, in this trial, the traditional clinical endpoint of PFS failed to demonstrate a benefit of adding reparixin to paclitaxel in advanced TNBC patients. However, this does not mean that CSC-targeting agents may not have a future role in treatment, as there are possible explanations for this negative result. First, two groups may have been unbalanced for presence of CSC in the metastatic tumor, with reparixin arm having more CSC which convey a worse prognosis [27, 28]. Second, targeting a single CSC survival pathway may not be sufficient. Lastly, only a proportion of ALDH + CSC express CXCR1 at any time point [4] and the clinical schedule of reparixin (21 days followed by 7 days off each 28-day cycle) different than the preclinical administration for 28 consecutive days [4] may have allowed CSC survival. Future CSC studies would benefit of readily available tumor tissue (e.g., breast cancer patients undergoing neoadjuvant treatment) to perform correlative studies with adequate number of sufficient samples from all treatment groups.

## Supplementary Information

Below is the link to the electronic supplementary material.

Supplementary file1 (DOCX 228 kb)

Supplementary file2 (DOCX 19 kb)

## Data Availability

All data presented can be found in the Clinical Study Report available at Dompé farmaceutici s.p.a., Milano, Italy.
