# Supplementary material for: A randomized, placebo-controlled phase 2 study of paclitaxel in combination with reparixin compared to paclitaxel alone as front-line therapy for metastatic triple-negative breast cancer (fRida)
Source: Breast Cancer Res Treat. 2021 Sep 3;190(2):265–75. doi: 10.1007/s10549-021-06367-5 (PMC8558154; doi:10.1007/s10549-021-06367-5)

**Figure S1. Progression-free survival and overall survival in the ALDH1^+^ population.** Kaplan-Meier estimates of PFS according to RECIST 1.1 and IRR evaluation (**A**) and OS (**B**) among subjects in the ITT population with ALDH1^+^ CSC in metastatic tissue. Stratified hazard ratios for disease progression or death (PFS) and death (OS) are reported along with *p* values. Tick marks indicate censored data.

**Figure S2**. **Progression-free survival and overall survival in the CD24^-^/CD44^+^ population.** Kaplan-Meier estimates of PFS according to RECIST 1.1 and IRR evaluation (A) and OS (B) among subjects in the ITT population with CD24^-^/CD44^+^ CSC in metastatic tissue. Stratified hazard ratios for disease progression or death (in analysis of PFS) or death (in analysis of OS) are reported along with *p* values. Tick marks indicate censored data.

**Figure S1**. ALDH-1+

**A**


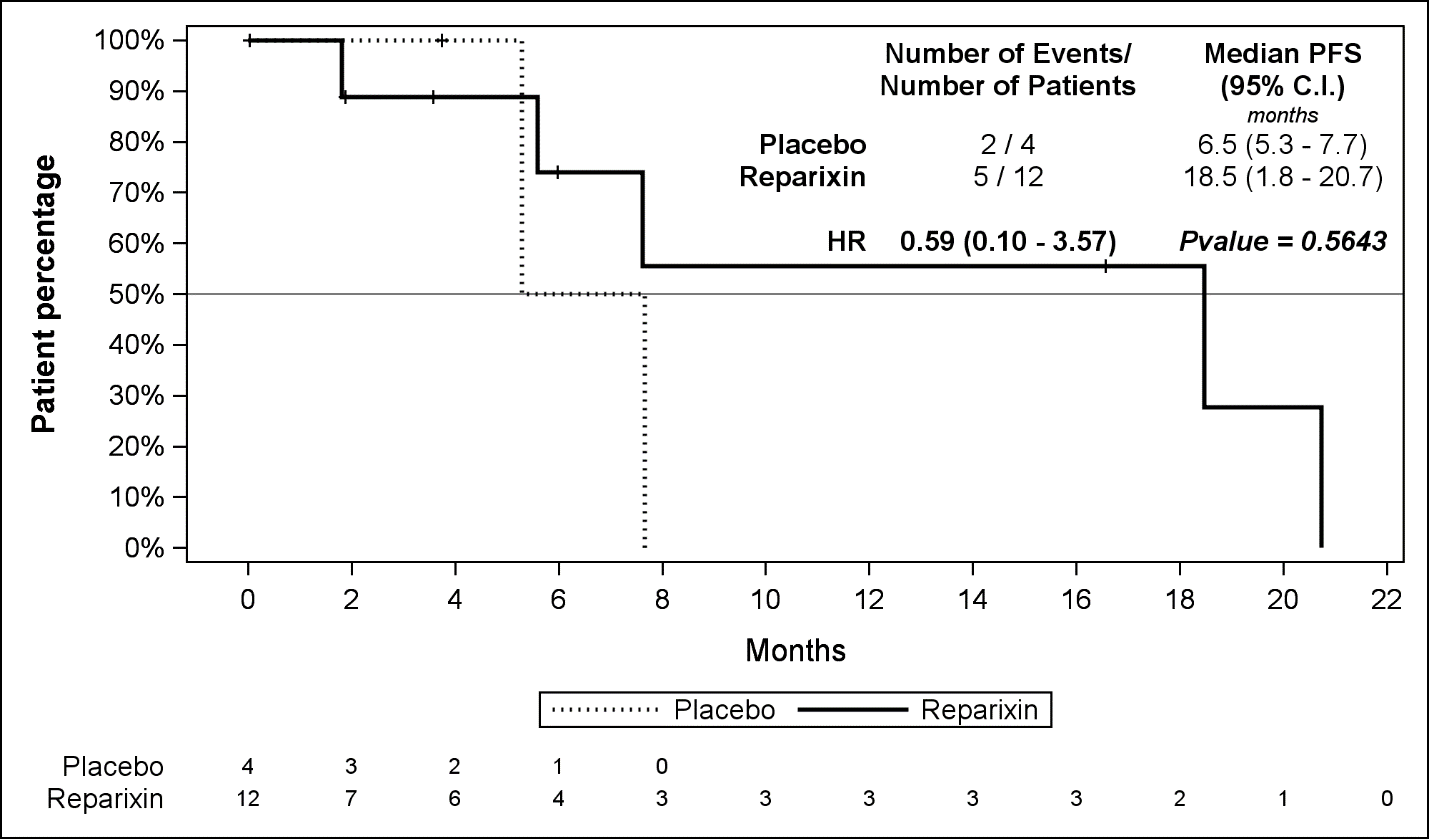


**B**


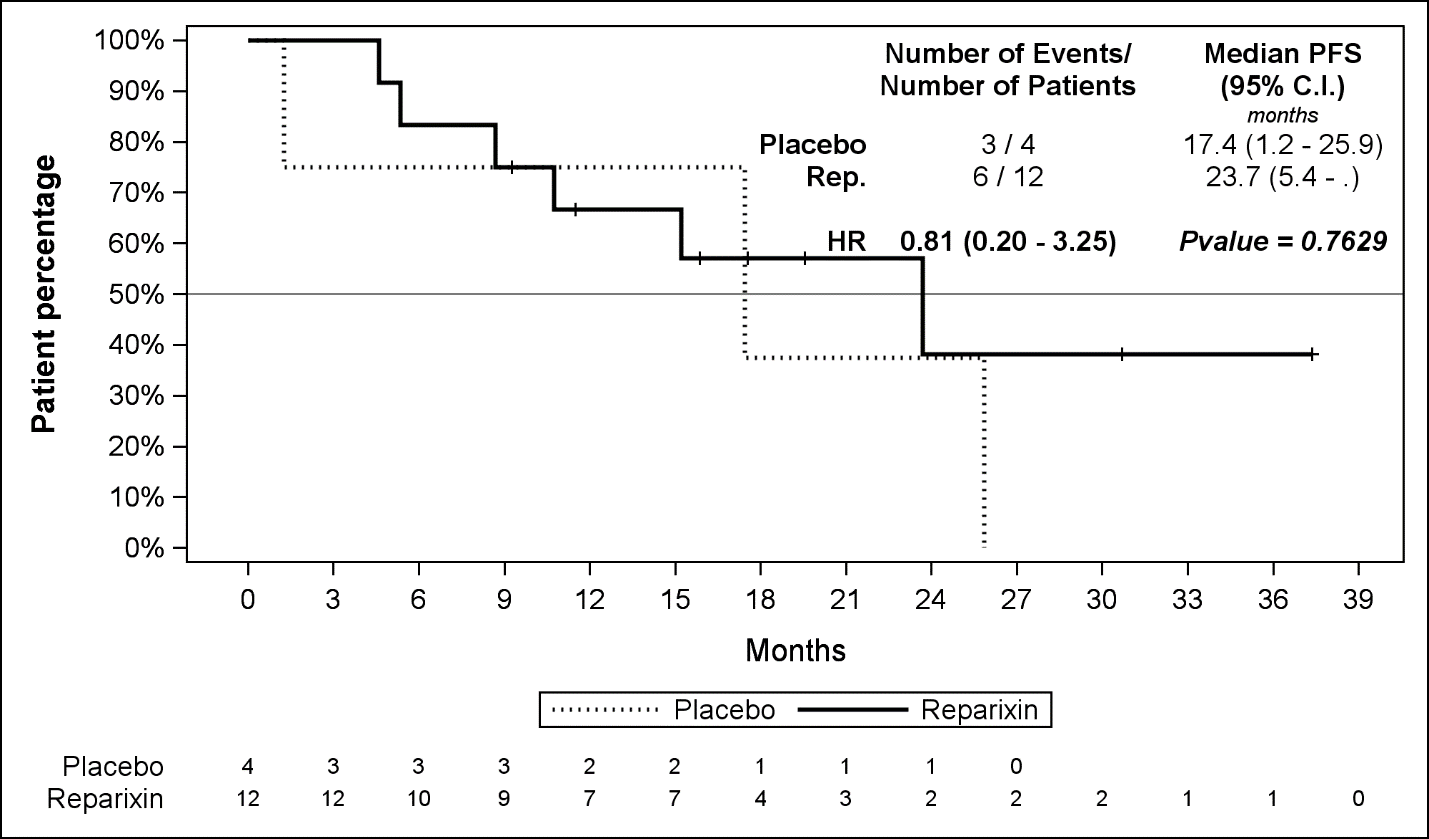


**Figure S2**. CD24-/CD44+

**A**
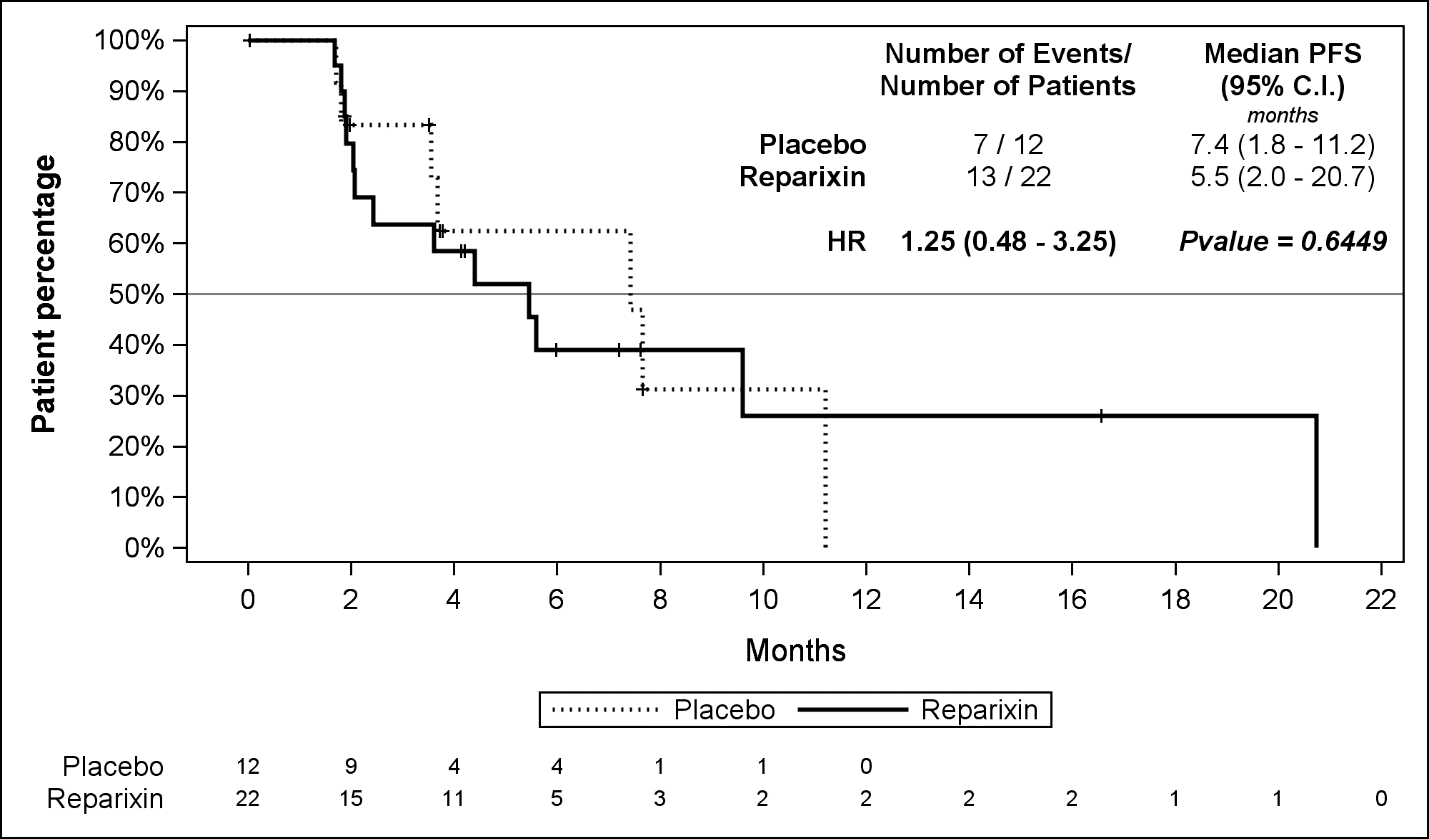


**B**


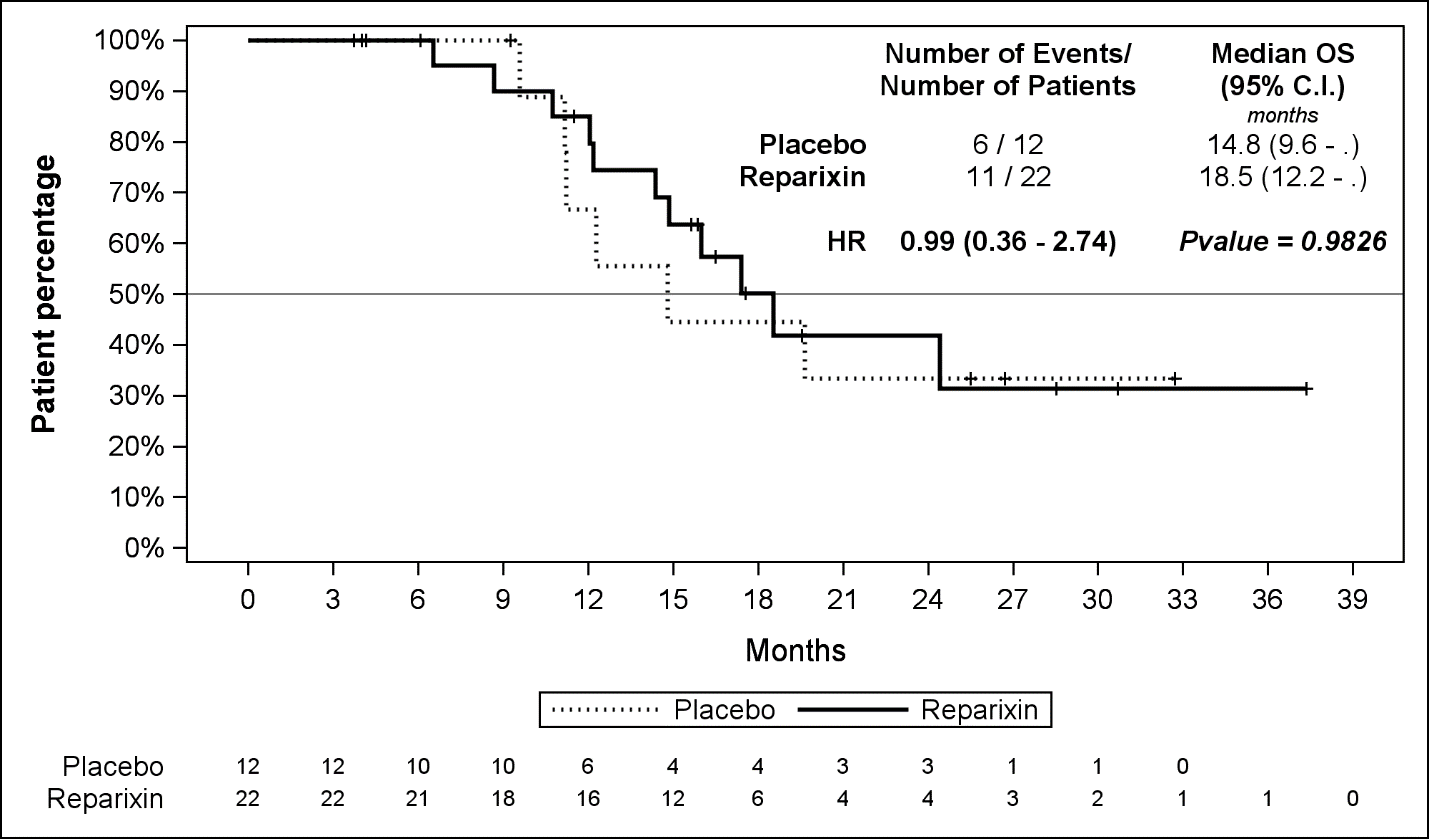

Supplement: Supplementary file 1 — Supplementary file1 (DOCX 228 kb) [file 10549_2021_6367_MOESM1_ESM.docx]
