# Supplementary material for: A randomized, placebo-controlled phase 2 study of paclitaxel in combination with reparixin compared to paclitaxel alone as front-line therapy for metastatic triple-negative breast cancer (fRida)
Source: Breast Cancer Res Treat. 2021 Sep 3;190(2):265–75. doi: 10.1007/s10549-021-06367-5 (PMC8558154; doi:10.1007/s10549-021-06367-5)
Supplement: Supplementary file 2 — Supplementary file2 (DOCX 19 kb) [file 10549_2021_6367_MOESM2_ESM.docx]

**Table S1**.

**Table S1**. **Cancer systemic therapies received during study follow-up (safety population)**.

| **subjects with indicated**  **treatment – n (%)** | **reparixin + paclitaxel**  **n=61** | | **placebo + paclitaxel**  **n=60** | | **total**  **n=121** |
| --- | --- | --- | --- | --- | --- |
|  | | | | | |
| **chemotherapy*** | | | | | |
| capecitabine | 24 (39.3) | 17 (28.3) | | 41 (33.9) | |
| eribulin | 9 (14.8) | 9 (15.0) | | 18 (14.9) | |
| carboplatin+gemcitabine | 8 (13.1) | 8 (13.3) | | 16 (13.2) | |
| vinorelbine | 6 (9.8) | 7 (11.7) | | 13 (10.7) | |
| cyclophosphamide | 9 (14.8) | 2 (3.3) | | 11 (9.1) | |
| epirubicin | 4 (6.6) | 2 (3.3) | | 6 (5.0) | |
| **immunotherapy** | | | | | |
| pembrolizumab | 1 (1.6) | 3 (5.0) | | 4 (3.3) | |
| atezolizumab | 0 | 2 (3.3) | | 2 (1.7) | |
| sacituzumab govitecan | 1 (1.6) | 1 (1.7) | | 2 (1.7) | |
| **hormonal therapy** |  | |  | |  |
| bicalutamide | 1 (1.6) | 0 | | 1 (0.8) | |
| enzalutamide | 1 (1.6) | 0 | | 1 (0.8) | |
| anastrozole | 0 | 1 (1.7) | | 1 (0.8) | |
| **antiangiogenic** | | | | | |
| bevacizumab** | 0 | 5 (8.3) | | 5 (4.1) | |
| **PARP inhs.** | | | | | |
| niraparib | 1 (1.6) | 0 | | 1 (0.8) | |
| olaparib | 0 | 1 (1.7) | | 1 (0.8) | |
| **investigational agents** | | | | | |
| undisclosed | 2 (3.3) | 4 (6.6) | | 6 (5.0) | |

* in at least 5% of safety population in either arm

** single agent and combination therapies
